# Solid Lipid Nanoparticles Produced via a Coacervation Method as Promising Carriers for Controlled Release of Quercetin

**DOI:** 10.3390/molecules26092694

**Published:** 2021-05-04

**Authors:** Luigi Talarico, Marco Consumi, Gemma Leone, Gabriella Tamasi, Agnese Magnani

**Affiliations:** 1 Department of Biotechnology, Chemistry and Pharmacy, University of Siena, Via Aldo Moro 2, 53100 Siena, Italy; luigi.talarico@student.unisi.it (L.T.); gemma.leone@unisi.it (G.L.); gabriella.tamasi@unisi.it (G.T.); 2 National Interuniversity Consortium of Materials Science and Technology (INSTM)—Siena Research Unit, Via G. Giusti 9, 50121 Firenze, Italy

**Keywords:** solid lipid nanoparticles, Quercetin, coacervation, FTIR, ToF-SIMS, drug delivery, controlled release, antioxidant

## Abstract

Quercetin is a poorly water-soluble flavonoid with many benefits to human health. Besides the natural food resources that may provide Quercetin, the interest in delivery systems that could enhance its bioavailability in the human body has seen growth in recent years. Promising delivery system candidates are represented by Solid Lipid Nanoparticles (SLNs) which are composed of well-tolerated compounds and provide a relatively high encapsulation efficiency and suitable controlled release. In this study, Quercetin-loaded and negatively charged Solid Lipid Nanoparticles were synthesized based on a coacervation method, using stearic acid as a core lipid and Arabic Gum as a stabilizer. Samples were qualitatively characterized by Dynamic light scattering (DLS), Zeta Potential, Surface infrared spectroscopy (FTIR-ATR), and Time of flight secondary ion mass spectrometry (ToF-SIMS). Encapsulation efficiency, drug release, and antioxidant effect against ABTS^•+^ were evaluated in vitro by UV–VIS spectrophotometry.

## 1. Introduction

The beneficial properties of many foods are correlated to the consumption of various compounds that are not enlisted in the main nutrients, those compounds, called nutraceuticals, can help in the prevention of diseases, and they have an overall good effect on health. As part of a diet, flavonoids are usually assimilated by the organism in the small intestine [1] and their rate of absorption may depend on their structure, solubility, molecular size, and pKa [2,3].

Quercetin (Querc; 3, 3′, 4′, 5, 7—pentahydroxyflavone) is a polyphenolic compound found in onions, red wine, and green tea [4,5]. This nutraceutical, besides having anticancer activity and both antioxidant and pro-oxidant activity—depending on the cells’ redox state and flavonoid concentration [6,7]—shows other significant properties, such as neuroprotective, anti-inflammatory [8,9], antimicrobial, and antiallergic activity [10]. It was reported as an asthma inhibitor if inhaled [11], and it is also potentially used for the prevention and treatment of SARS-CoV-2 [12,13] and as a general antiviral, as Quercetin inhibits rhinovirus replication both in vitro and in vivo [6]. In this context, any formulation that can be useful to improve the absorption of Quercetin by an organism could be considered relevant [14]. Furthermost, Quercetin very quickly degrades in alkaline environments, and for this reason, it is not practical to add more Quercetin to processed food products, because it will result in a diminution of shelf life [15,16,17].

Although Quercetin has mainly positive effects on human health, side effects accompany the assumption of high doses of this polyphenol. If consumed in high doses, Quercetin may cause DNA damage, mutagenesis, and genotoxicity, even if those effects are not proven by in vivo studies [8]. However, the clinical application of this nutraceutical is negatively affected by its poor solubility in aqueous media, rapid clearance from the body, and low intrinsic activity [18].

Suitable carriers based on hydrogels [19,20], polysaccharide complexes [21], and liposomes [22,23,24] have been designed as delivery systems of poorly soluble nutraceuticals allowing their transport in aqueous media and in situ delivery, while avoiding possible undesired side effects. SLNs show greater biocompatibility, lower toxicity, and better delivery of lipophilic drugs with respect to hydrogel. Furthermore, SLNs are more physically stable with respect to liposomes and they show a favorable cost-effective ratio.

Solid Lipid Nanoparticles (SLNs) are one of the promising nanocarriers for both hydrophilic and lipophilic compounds, because of their low toxicity, physical stability [25], high encapsulation efficiency [26], and the ability to modulate the drug release kinetics. They are generally composed of a lipidic solid core and a stabilizing environment that improves physical stability during storage and reduces the interfacial energy between the lipidic core and aqueous environments [27,28].

**Figure 1:**
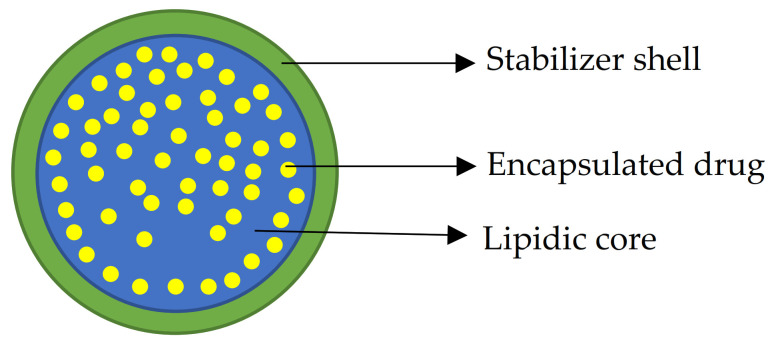
Schematic representation of loaded Solid Lipid Nanoparticles: a lipidic core (blue) with loaded drug (yellow) and a stabilizer outer shell (green).

Because of their easy dispersion in water, SLNs are also appropriate for intravenous administration, and the possibility of surface functionalization makes them a suitable carrier for theragnostic treatments [29]. Due to their solid and crystalline nature, lipids in the SLN can be packed in different conformations that could affect drug entrapment, and that is why proper storage and treatment are necessary to avoid drug expulsion from solid lipid nanoparticles; the less packed lipid’s crystalline form obtained during the synthesis tends to evolve into more packed and stable forms, resulting in drug expulsion and particle agglomerates [30]. Figure 1 represents the general structure of Solid Lipid Nanoparticles.

The most common methods to obtain solid lipid nanoparticles are ultrasonication [31], high-pressure homogenization [32], microemulsion [33], and acoustic cavitation [34]. Many of these methods are usually time-consuming and require specific instrumentation to be performed. Coacervation [35,36,37,38] is a simple method based on the heating of aqueous solutions with a specific salt of fatty acids above its *Krafft point*. Heating the lipid’s solution is one of the crucial points of this method, as the *Krafft Point* is defined as the temperature above which the solubility of a surfactant becomes equal to the critical micellar concentration [39]. The emulsion is then created adding the stabilizer solution, and a coacervating agent to trigger the SLN formation by pH change. This mixture is then quickly refrigerated and always kept under stirring. Regarding the drug distribution within the solid lipid nanoparticles, three main possibilities have been demonstrated: a drug-enriched core, a drug-enriched shell, and a homogeneous matrix. Those distributions are difficult to be discerned with common techniques [26], but they usually discriminate the release profiles. Although many Solid Lipid Nanoparticles loaded with Quercetin have been already studied [40,41,42,43,44,45], there are not so many examples of loaded SLNs prepared with a coacervation method. In this work, this method was used to synthesize SLN loaded with Quercetin. Loaded and unloaded SLNs were qualitatively analyzed using FTIR-ATR, ToF—SIMS, DLS, and Zeta potential. Quercetin encapsulation efficiency, in vitro release, and QuercSLN antioxidant activity against ABTS^•+^ were evaluated using UV—VIS spectrophotometry methods.

## 2. Results and Discussions

### 2.1. Coacervation Method for Quercetin Loaded Solid Lipid Nanoparticles

To prepare empty and loaded SLNs, the coacervation procedure was adopted and the different components used to synthesize Quercetin loaded and unloaded SLNs are listed in Table 1.

**Figure 2:**
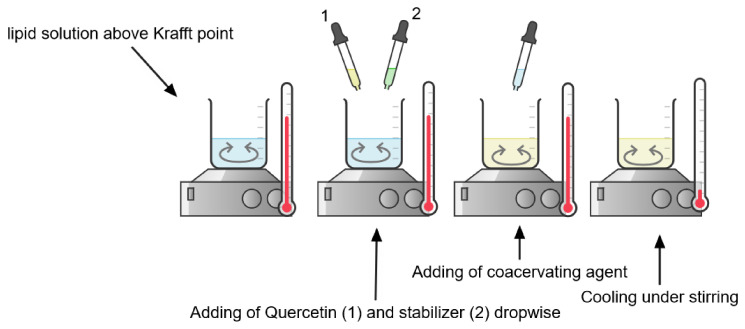
Steps of coacervation process for QuercSLN (Created with Chemix).

During the coacervation procedure, the surfactant solution was heated above the *Krafft Point* under stirring. In the case of loaded SLNs, the nutraceutical compound was added after this step. Then, the stabilizer and coacervating agent were added dropwise. Once pH ≈ 4 was reached, the solution became opalescent, and the temperature was maintained at 15 °C. The dispersion was immediately centrifuged to remove unentrapped Quercetin. Figure 2 shows the coacervation process for QuercSLNs preparation

### 2.2. DLS and Zeta Potential Measurements

The loaded SLNs showed a diameter of 480. 1 ± 112.0 nm with a PDI 0.182 ± 0.101 and a Zeta-potential of −27.4 ± 0.6 mV. Both these data were obtained on a diluted suspension (10% *v*/*v* QuercSLN suspension).

Regarding the purification process by filtration, particle size and zeta potential were evaluated also for particles that can pass through a 0.2 µm filter, and for a solution obtained from SLNs of larger dimensions recovered from it. Table 2 summarizes the results of Zeta potential and size analysis. Size distributions graphs are available in SI (Figures S1–S6).

The negative Zeta potential is given by the stabilizer shell composed of Arabic gum, probably because of either the polymer disposition [36] or the presence of deprotonated d-glucuronic acid and 4-*O*-methyl–d–glucuronic acid [46], in the conditions of analysis. Moreover, peptides and proteins that are present in this natural mixture, may also contribute to the net surface charge of SLNs.

### 2.3. FTIR-ATR Analysis

**Figure 3:**
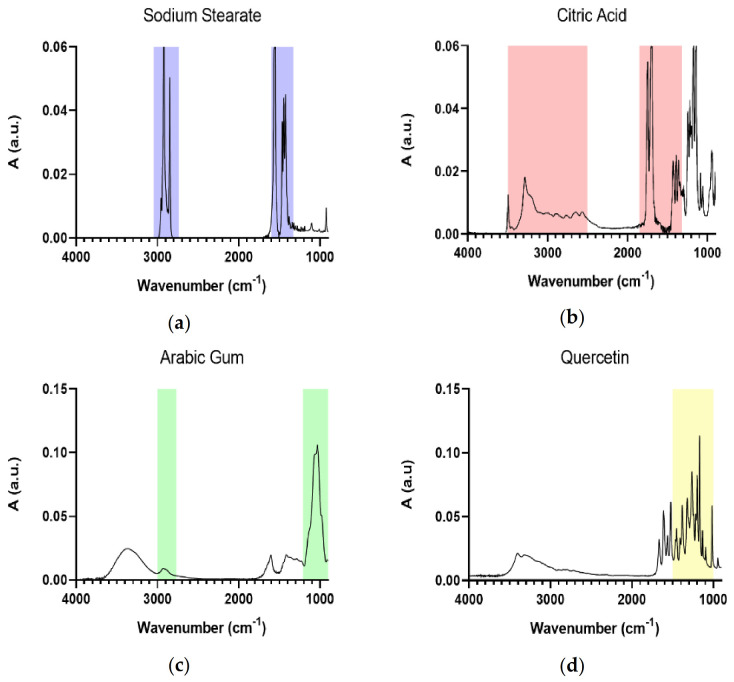
IR spectra of SLNs’ main components: Sodium Stearate (**a**), Citric Acid (**b**), Arabic Gum (**c**), and Quercetin (**d**). Distinctive signals are highlighted.

Figure 3 shows the IR spectra of the principal components of Solid Lipid Nanoparticles. Table 3 summarizes the main wavenumbers observed in the IR spectra together with their assignments.

The IR spectrum of Sodium Stearate (Figure 3a) is characterized by the intense CH_2_ signal between 3000 and 2760 cm^−1^, due to symmetric and asymmetric stretching, and for the COO^−^ absorptions at 1600 and 1400 cm^−1^ (asymmetric and symmetric stretching, respectively). The IR spectrum of Citric acid (Figure 3b) shows the broad OH band between 4000 and 3000 cm^−1^, with the broadening effect mainly due to H-bonds formation, and the COOH stretching at 1700 cm^−1^. For Arabic gum, the IR spectrum (Figure 3c) is dominated by the CH_2_ band between 3000 and 2800 cm^−1^, and the polysaccharidic distinctive signals due to the vibration of the polysaccharidic rings in the 1100–900 cm^−1^ range. Finally, the main distinctive signals for Quercetin (Figure 3d) fall in the 1600–1000 cm^−1^ range, due to C=C aromatic bonds (1520 cm^−1^), =C-OH phenol group (1321 cm^−1^), typical phenolic moiety (1377 cm^−1^), C-O-C stretching (1260 cm^−1^), and the conjugation of aryl ether with C=C-O of the aromatic ring (1014 cm^−1^) [47]. Moreover, the broad band of OH stretching is observed in the 3402 and 3324 cm^−1^ spectral range.

**Figure 4:**
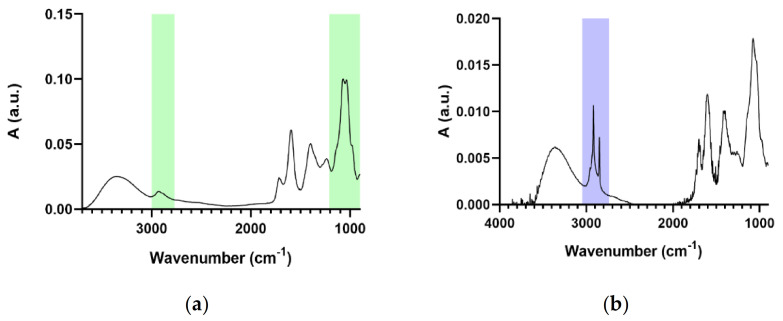
IR spectra of filtered (**a**) and recovered (**b**) SLNs solution.

Figure 4 shows the IR spectra of the suspension of SLNs. In particular, the IR spectrum of the SLNs portion passed through the filter is shown in Figure 4a, whereas Figure 4b shows the IR spectrum of SLNs recovered from the 0.2 µm filter surface (they represent the SLNs portion that does not pass through the filter because of higher dimensions). The distinctive signals in these figures are highlighted with the same color as the compounds that generate them.

It is noticeable how the filtration process let pass through the 0.2 µm pores only Solid Lipid Nanoparticles with a core structure (stearic acid) that is smaller in proportion to the stabilizer (Arabic gum) shell, while the portion obtained from the residues on the filter surface shows how SLNs that cannot pass through the pores had a stearic acid core that is bigger in proportion to the Arabic gum stabilizing shell.

**Figure 5:**
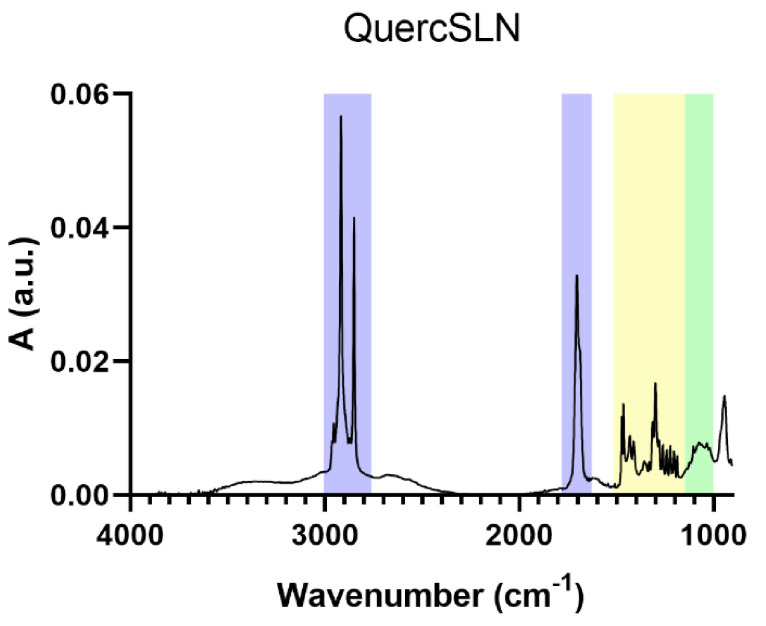
IR spectrum of QuercSLN.

The IR spectrum of QuercSLNs is reported in Figure 5. It shows all the characteristic absorption bands of the main components. The presence of the absorption band centered at 1700 cm^−1^, assigned to the protonated carboxylic group (COOH), and the absence of the two absorptions of the carboxylate group (COO^−^) (1600 and 1400 cm^−1^), confirm that the stearate is in the form of stearic acid.

### 2.4. ToF-SIMS

Samples of empty SLNs and Quercetin loaded-SLNs were analyzed by ToF-SIMS to deeply analyze their composition. Both samples were mainly composed of stearic acid and Arabic Gum, with characteristic fragments ions listed in Table 4. Arabic Gum is a mixture of primarily l—arabinose, l—rhamnose, d—glucuronic acid, and 1—3 β—galactopyranosyl; those monosaccharides fragmentations and molecular ions [48] are used as a reference for the Arabic gum presence. Fragments at *m*/*z* 123 and 125 were present in both spectra, and they could be assigned to the fragmentation of both stearic acid and Quercetin, thus these two fragments were not included as indicatives of Quercetin presence in the loaded SLNs.

**Figure 6:**
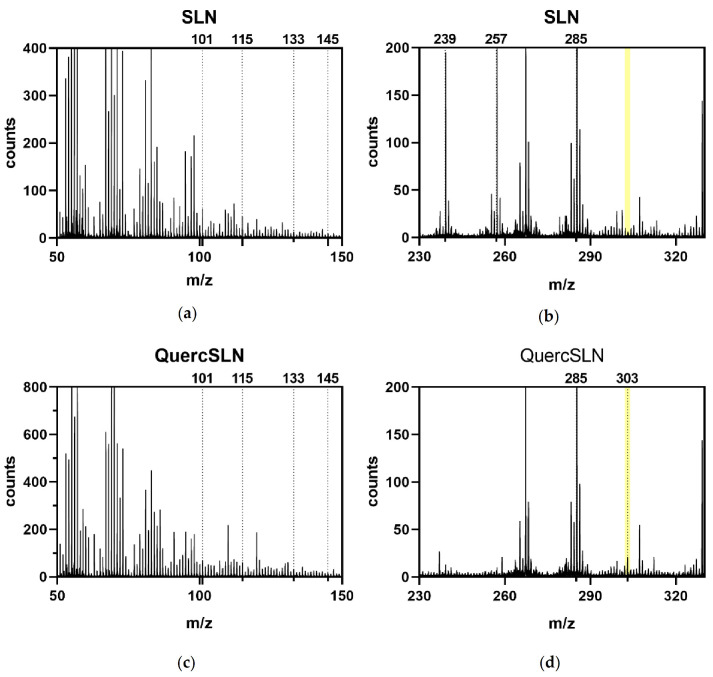
ToF-SIMS (positive ions) spectra extracts of SLNs (**a**,**b**), and QuercSLNs (**c**,**d**).

Figure 6 reports spectral extracts from both bare Solid Lipid Nanoparticles and Quercetin-loaded SLNs. Only the QuercSLNs spectrum shows the characteristic peak of Quercetin molecular ion at 303 *m*/*z*.

### 2.5. Drug Encapsulation Efficiency

The amount of free Quercetin in QuercSLN suspension was determined by interpolation of samples’ absorbances obtained from centrifugation with a calibration curve in the range 1 × 10^−6^–5 × 10^−5^ M (R^2^ = 0.9997) (Figure S7). The formulation for QuercSLN showed an encapsulation efficiency of 80.4 ± 0.26%calculated using Equation (1).

### 2.6. In Vitro Drug Release

**Figure 7:**
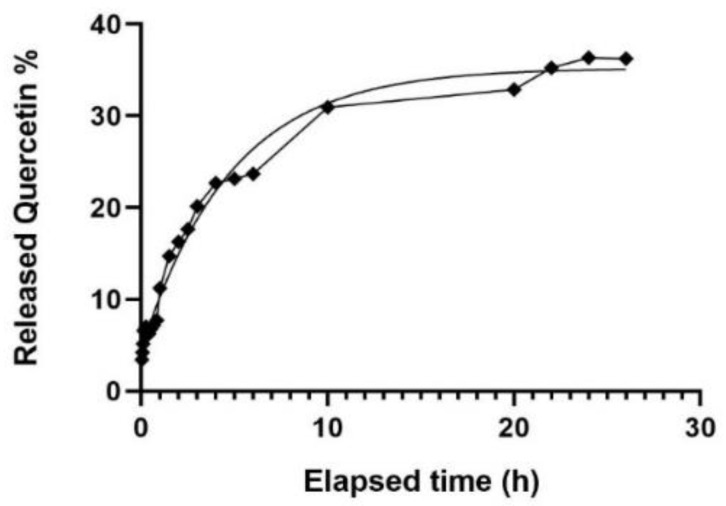
Quercetin release from QuercSLN in 65:35 H_2_O/EtOH dissolution medium. Table A1 reports values ± SD.

The release profile of Quercetin in a 65:35 H_2_O/Ethanol dissolution medium is reported in Figure 7. Quercetin release from QuercSLN in 65:35 H2O/EtOH dissolution medium. Table A1 reports values ± SD.

The release fits an exponential curve with equation Y = 35.22 ∗ (1 − exp(−0.2083×) (R^2^ = 0.9876). The total amount of Quercetin released through the membrane in the dissolution medium after 26 h is 36.7% of the total Quercetin used during the synthesis.

In the first 4/5 min, ≈20% of the encapsulated Quercetin is released. This amount corresponds to approximately half of the total amount released within 26 h (≈37% of encapsulated). The initial faster release suggests that ≈50% of the released Quercetin may be located within the stabilizer shell (the outermost layer of the nanoparticles). The remaining amount is released much more slowly within the 26 h, suggesting that this quantity (≈50% of the total released) could be entrapped in the core of SLNs. These findings suggest that Quercetin could be homogeneously distributed through the overall nanoparticle structure (≈50% in the nucleus and the other ≈50% in the outer shell of the SLNs).

### 2.7. Antioxidant Quercetin-Loaded SLNs Assay

Antioxidant activity was measured in vitro by an ABTS^•+^ assay and compared to that of a free Quercetin solution of the same concentration to understand if and to what extent the nanocarrier could affect the scavenging effect of the Quercetin and to assess the SLNs effect in controlling the nutraceutical delivery over time. The composition of the samples for the evaluation of the antioxidant activity of QuercSLN is shown in Table 5.

**Figure 8:**
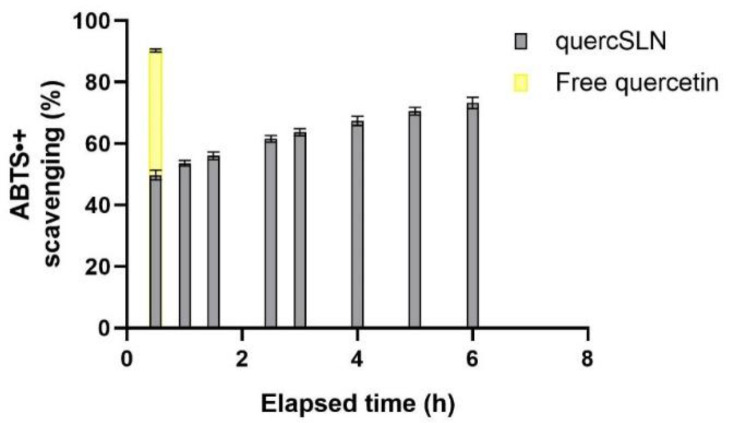
Time-dependent antioxidant activity of QuercSLN compared to that of a free Quercetin solution of the same concentration.

The absorbances of samples were measured at regular intervals within 6 h after preparation, with a total scavenging effect after 6 h of ≈73.0%. After 24 h the solution of Quercetin-loaded SLNs was completely discolored. The same test performed on 10 µL of a 1.6-mM Quercetin solution provided a 90.3 ± 0.5% scavenging effect after 30 min from sample preparation. This demonstrated the preserved antioxidant activity of Quercetin in QuercSLNs and its time-persistent effect due to a controlled release of the antioxidant molecule. The time-dependent behavior of the analyzed samples is reported in Figure 8.

## 3. Materials and Methods

### 3.1. Materials

Citric acid 99%, Sodium Stearate, Quercetin (3, 3′, 4′, 5, 7—pentahydroxyflavone) dihydrate, Pur-A-Lyzer™ Mega 3500 kit MWCO 3.5 kDa membranes, ABTS (2,2′-Azino-bis (3-ethylbenzthiazoline-6-sulfonic acid)), and K_2_S_2_O_8_ (Potassium persulfate) were purchased from Merk Italy Milano, (Italy). Instant soluble Gum Acacia 396I (Arabic Gum) was purchased from Alland & Robert Port-Mort, (France).

### 3.2. Preparation of Quercetin-Loaded Solid Lipid Nanoparticles

The coacervation method proposed by Battaglia et al. [36,49] was adopted to obtain negatively surface-charged Solid Lipid Nanoparticles. The lipid’s salt (sodium stearate) was dispersed in distilled water and heated above the *Krafft Point* under a 300 rpm stirring until a clear solution was obtained. A Quercetin solution (5 mM) in absolute ethanol was then added to the hot lipid solution. Then the stabilizer solution (Arabic gum) was added dropwise to the hot mixture under stirring. Finally, the coacervating agent (Citric acid) is added dropwise until pH 4 was reached, and an iced water bath was used to bring the temperature down to 15 °C while keeping the mixture under stirring. Non-loaded SLNs were prepared following the same procedure, without adding the Quercetin solution.

### 3.3. Purification of SNLs

The preparations were purified by filtration with a 0.2 µm syringe filter. In particular, the QuercSLNs suspension was centrifuged to separate unencapsulated Quercetin (precipitate) from the nanoparticle suspension and then filtered. The obtained SLN were stored at 4 °C until their use.

### 3.4. Quercetin Encapsulation Efficiency

The free amount of drug in the freshly prepared QuercSLNs suspension was separated by centrifugation [42,50] at 4000 rpm for 10 min. Due to its high insolubility in water, the excess amount of Quercetin precipitates. The supernatant with SLNs suspension is then separated and the precipitated Quercetin pellets were resuspended in ethanol for determination via UV–VIS measurements of Encapsulation Efficiency, reading the absorbances at 375 nm and then calculated by Equation (1).

(1) $$EE\left(\%\right)=\frac{total\;quercetin\;added-free\;quercetin}{total\;quercetin\;added}\times 100$$

All the UV–VIS measurements were carried out in triplicate using a Perkin–Elmer Lambda 25 spectrophotometer.

### 3.5. Characterization of QuercSLNs

#### 3.5.1. DLS and Zeta Potential Measurements

Particle size and Zeta potential were determined with Photon Correlation Spectroscopy on a Malvern Zetasizer Nano ZS-90 at 25 ° C and a 90° detector angle. DLS measurements were performed on a diluted (10% *v*/*v*) SNLs suspension before and after filtration with 0.2 µm syringe filter to check the homogeneity of particle size. Measurements were conducted in triplicate.

#### 3.5.2. FTIR-ATR Measurements

FTIR spectra of loaded and unloaded SLNs were recorded with a Nicolet iS50 FT-IR using an ATR accessory equipped with ZnSe crystal as IRE. FT-IR was primarily used to determine the composition of both Quercetin-loaded and unloaded Solid Lipid Nanoparticles. To investigate the role of a filtration step in the purification process (see Section 2.3) spectra of collected SLN and filtrate were recorded.

#### 3.5.3. ToF-SIMS Analysis

ToF-SIMS experiments were carried out on a TRIFT III spectrometer (Physical Electronics, Chanhassen, MN, USA) equipped with a 22 KeV Au^+^ gold liquid-metal primary ion source with a beam current of 600 pA at an incidence angle of 45°. The gold source was used for both sputtering and analysis. SLNs were analyzed on Si substrates after desiccation in a vacuum box. Substrates were maintained overnight in a conditioning pre-chamber with a vacuum value of 10^−4^ Pa and then transferred to the analyzing chamber at a vacuum value of 10^−9^ Pa without further manipulation. For the analysis, positive and negative ion spectra were acquired with a pulsed, bunched primary ion beam, by rastering the ion beam over a 200 × 200 µm sample area and maintaining static SIMS conditions (primary ion dose density less than 10^12^ ions/cm^2^). Positive ion spectra were calibrated with CH_3_^+^ (*m*/*z* 15.023), C_2_H_3_^+^ (*m*/*z* 27.023), C_3_H_5_^+^ (*m*/*z* 15.023) fragments, as reported elsewhere [51]. The mass resolution (m/ΔM) was 3000 at *m*/*z* 27.

### 3.6. In Vitro Quercetin Release

In vitro Quercetin release was performed using a 3.5 kDa MWCO dialysis bag soaked overnight in distilled water for conditioning. The bag was emptied and then filled with 4.5 mL of QuercSLNs suspension and soaked in 40 mL of 65:35 H_2_O/Ethanol mixture used as releasing medium. As reported in literature [45], adding Ethanol to the dissolution medium improves the solubility of Quercetin. The system was kept at 37 °C using a thermostated water bath, under gentle magnetic stirring. Aliquots of 2 mL of the dissolution method were withdrawn at specific time intervals and to provide sink conditions the same amount of fresh releasing medium was added to the system. The aliquots were then analyzed by UV–VIS spectrophotometry, reading the absorbances at 375 nm, as previously described for the determination of unentrapped Quercetin. The calibration curve is reported in Figure S7.

### 3.7. Antioxidant Activity

An antioxidant activity assay [52,53] was carried out with QuercSLNs to evaluate the reduction of activity against ABTS. For this method, ABTS was dissolved in water with a final concentration of 7.77 mM. The ABTS^•+^ radical was produced via activation with potassium persulfate and kept overnight in a dark place at room temperature. Before analysis, ABTS^•+^ was diluted with distilled water (1:30 *v*/*v*) and 10 µL QuercSLNs suspension was tested within 6 h reading the absorbance at 734 nm, using as blank the same amount of unloaded SLNs. Results were compared with data obtained from a Quercetin solution (1.6 mM) obtained following the same procedure but reading the absorbance value 30 min after preparation and using as blank an ABTS^•+^ solution. The scavenging effect percentage of QuercSLNs and Quercetin was evaluated using the following equation.

(2) $$scavenging\;\%=\left(1-\frac{A_{blank}}{A_{sample}}\right)\times 100$$

## 4. Conclusions

This study focused on the synthesis and characterization of Quercetin-loaded SLNs obtained via the coacervation method. This method was demonstrated as a high-loading method for Quercetin and an overall simple process to obtain solid lipid nanoparticles, as confirmed by both DLS and Zeta potential measurements that provided the dimensions and charge of the nanoparticles, and FTIR/ATR and ToF-SIMS that provided the proper SLNs composition.

The filtration process was successful in separating Solid Lipid Nanoparticles by their size, as confirmed by dimensional distributions given by Dynamic Light Scattering Infrared data. Moreover, Infrared analysis of the two SLNs portions (the filtered and the recovered from filter) have different composition with the filtered a lipid core smaller than the stabilizer shell and the recovered from filter showing a Stearic Acid core greater than the Arabic Gum stabilizing shell.

The release profile of QuercSLN followed an exponential plateau pathway within 26 h, evidencing a homogeneous distribution of the drug within the SLNs, and the system showed a controlled antioxidant effect compared to free Quercetin, demonstrating that the encapsulated nutraceutical preserves its antioxidant activity to a large extent (≈81% of that of free Quercetin).

Based on these data, Solid Lipid Nanoparticles synthesized via a coacervation method were demonstrated as suitable candidates as systems for the controlled delivery of Quercetin and other lipophilic drugs or nutraceuticals.

## Figures and Tables

**Table 1:** Amounts of components used for Solid Lipid Nanoparticles synthesis.

Components.	SLN	QuercSLN
Sodium stearate * (mg)	107	107
Arabic gum(mg)	100	200
1M Citric acid (mL)	0.2	0.2
Quercetin 5 mM (mL)	-	1.5
Water (mL)	9.8	9.8
Total volume (mL)	10	11.5

* 107 mg of sodium stearate corresponds to 100 mg of Stearic Acid.

**Table 2:** Particle size and Zeta potentials for QuercSLNs.

Sample	Size (Diameter) (nm)	PDI	Zeta Potential (mV)
QuercSLN	480.1 ± 112.0	0.182 ± 0.101	−27.4 ± 0.6
Filtered QuercSLN	279.4 ± 4.6	0.206 ± 0.013	−26.1 ± 0.3
Recovered from filter QuercSLN	422.0 ± 14.1	0.200 ± 0.029	−33.6 ± 0.4

**Table 3:** Main wavenumbers observed and relative assignment.

Wavenumber (cm^−1^)	Spectrum	Assignment
3000–2760	Sodium Stearate	CH_2_ symm. and asymm. stretching
1600		COO^−^ asymm. stretching
1400		COO^−^ symm. stretching
4000–3000	Citric Acid	OH group
1700		C=O stretching of COOH.
3000–2800	Arabic Gum	CH_2_ stretching
1100–900		Polysaccharidic signal
1520	Quercetin	C=C aromatic bonds
1321		=C-OH phenol group
1377		Phenolic moiety
1260		C-O-C stretching
1014		Aryl ether conjugation with C=C-O
3402, 3324		OH stretching

**Table 4:** Mass peaks assignment. Molecular ions are highlighted.

*m*/*z*	Sample	Assignment
285	SLN/QuercSLN	C_18_H_37_O_2_ + H^+^ (Stearic Acid)
267	SLN/QuercSLN	C_18_H_35_O (SA-H_2_O)
239	quercSLN	C_17_H_36_ (SA-COOH)
303	quercSLN	C_15_H_10_O_7_ + H^+^ (Quercetin)
91.05	quercSLN	C_7_H_7_^+^
101	SLN/quercSLN	C_5_H_9_O_2_^+^ (L—rhamnose)
115	SLN/quercSLN	M-2H_2_O + H (L—arabinose)
115	SLN/quercSLN	C2 to C6 fragment (D—galactose)
133	SLN/quercSLN	M-H_2_O + H (L—arabinose)
145	SLN/quercSLN	M-2H_2_O + H (L—rhamnose, D—Galactose)

**Table 5:** Samples compositions for antioxidant activity assay.

Sample	Blank	SLN	QuercSLN	Quercetin
SLN (µL)	-	10	-	-
QuercSLN (µL)	-	-	10	-
Quercetin 1.6 mM (µL)	-	-	-	10
ABTS^•+^ (mL)	1	1	1	1
Water (µL)	100	90	90	90
Total Volume (mL)	1.1	1.1	1.1	1.1

## Data Availability

Data sharing not applicable

## Supplementary Materials

The following are available online, Figure S1: Size distribution of Quercetin-loaded Solid Lipid Nanoparticles, Figure S2: Zeta potential distributions for Quercetin-loaded Solid Lipid Nanoparticles, Figure S3: Size distribution for filtered Quercetin-loaded Solid Lipid Nanoparticles, Figure S4 Zeta potential distributions for filtered Quercetin-loaded Solid Lipid Nanoparticles, Figure S5: Size distribution for recovered from filter Quercetin-loaded Solid Lipid Nanoparticles, Figure S7: Calibration curve for Quercetin in EtOH. R^2^ = 0.9997, eq. Y = 20630X − 0.009693.

## Appendix A

**Table A1:** Values and standard deviation of Quercetin release from QuercSLN.

Elapsed Time (h)	Value (%)
0:02:00	3.46 ± 0.09
0:04:00	4.26 ± 0.20
0:06:00	5.23 ± 0.06
0:08:00	6.66 ± 0.17
0:15:00	7.10 ± 0.15
0:20:00	6.28 ± 0.02
0:25:00	6.32 ± 0.05
0:30:00	6.84 ± 0.01
0:40:00	7.27 ± 0.03
0:50:00	7.83 ± 0.15
1:00:00	11.36 ± 0.02
1:30:00	14.93 ± 0.15
2:00:00	16.46 ± 0.06
2:30:00	17.9 ± 0.10
3:00:00	20.46 ± 0.06
4:00:00	23.0 ± 0.10
5:00:00	23.46 ± 0.06
6:00:00	24.0 ± 0.10
10:00:00	31.4 ± 0.20
20:00:00	33.3 ± 0.10
22:00:00	35.73 ± 0.06
24:00:00	36.83 ± 0.06
26:00:00	36.7 ± 0.10
