# Supplementary material for: Solid Lipid Nanoparticles Produced via a Coacervation Method as Promising Carriers for Controlled Release of Quercetin
Source: Molecules. 2021 May 4;26(9):2694. doi: 10.3390/molecules26092694 (PMC8125226; doi:10.3390/molecules26092694)
Supplement: Supplementary file 1 [file molecules-26-02694-s001.zip › molecules-1204684-supplementary.pdf]

Talarico, L.; Consumi, M.; Leone, G.; Tamasi, G.; Magnani, A.

Solid Lipid Nanoparticles produced via coacervation method as promising carriers for controlled release of Quercetin.

SUPPORTING INFORMATIONS

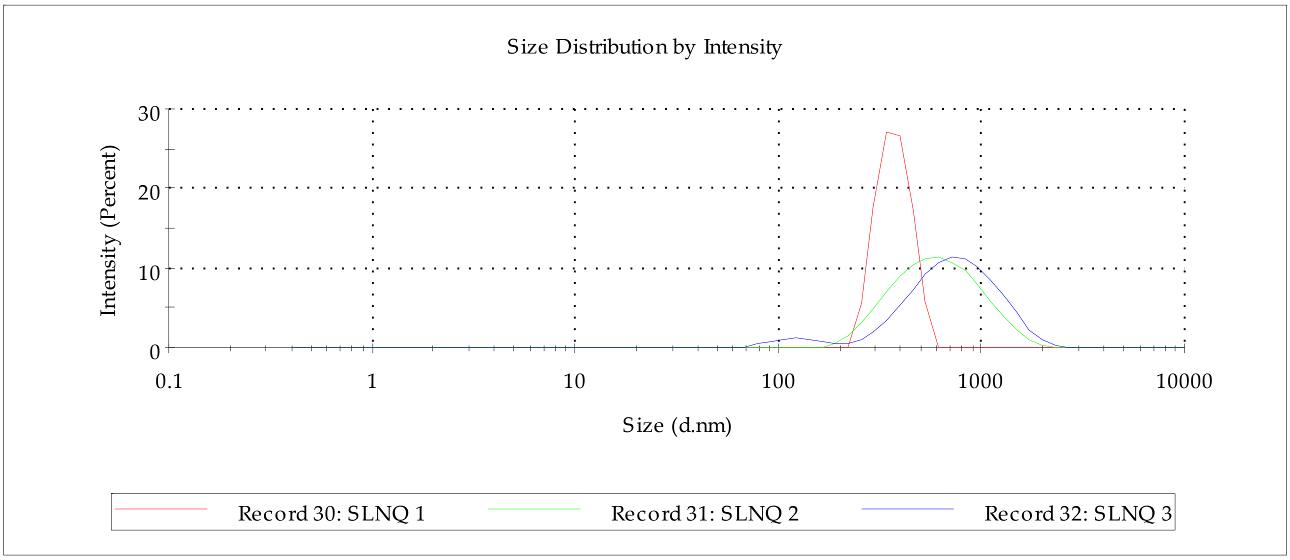

Figure S1: Size distribution of Quercetin-loaded Solid Lipid Nanoparticles

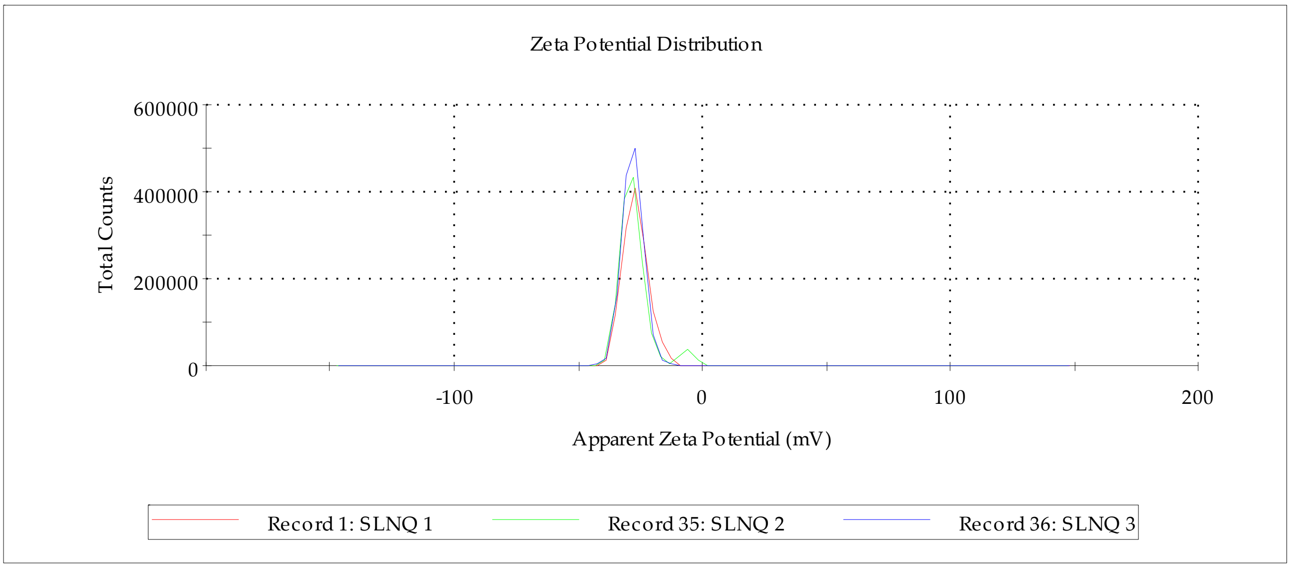

Figure S2: Zeta potential distributions for Quercetin-loaded Solid Lipid Nanoparticles

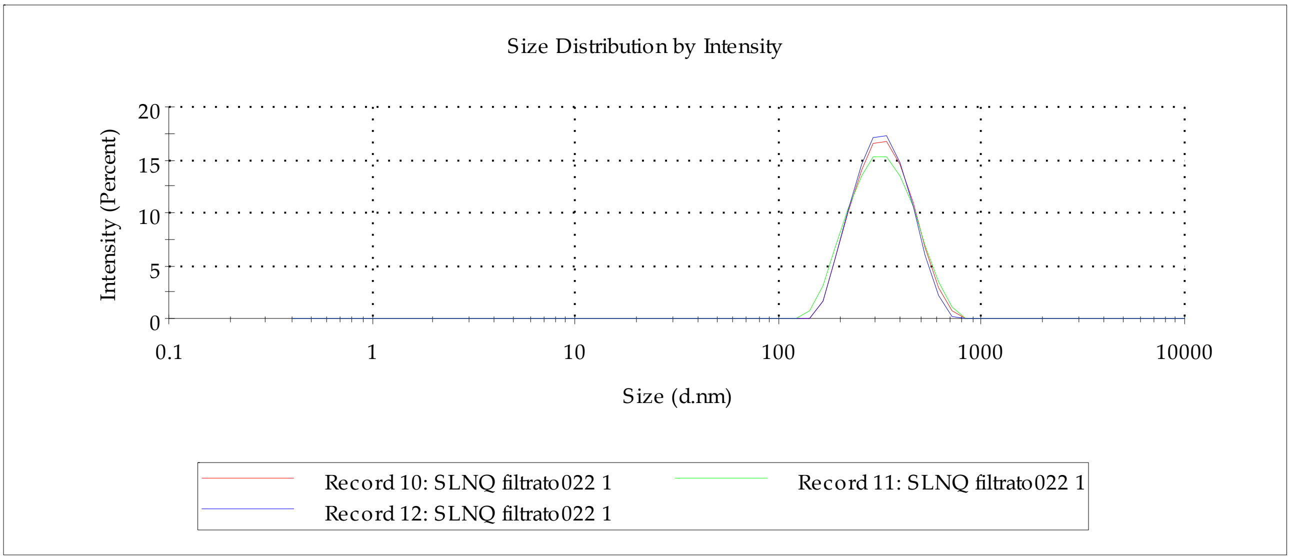

Figure S3: Size distributions for filtered Quercetin-loaded Solid Lipid Nanoparticles

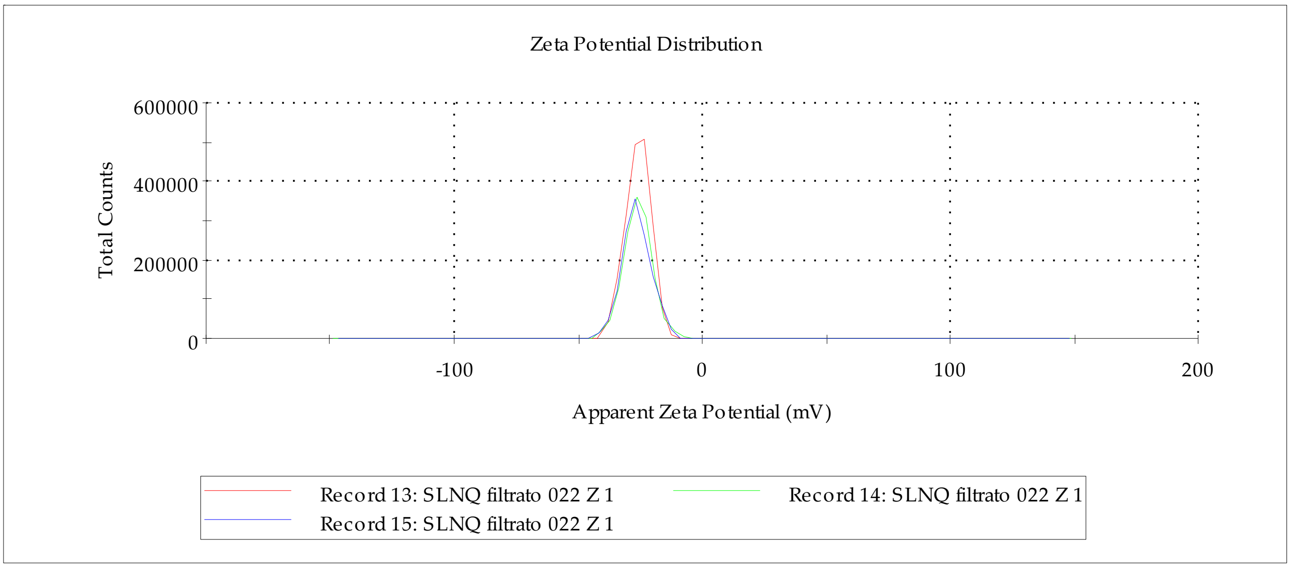

Figure S4: Zeta potential distributions for filtered Quercetin-loaded Solid Lipid Nanoparticles

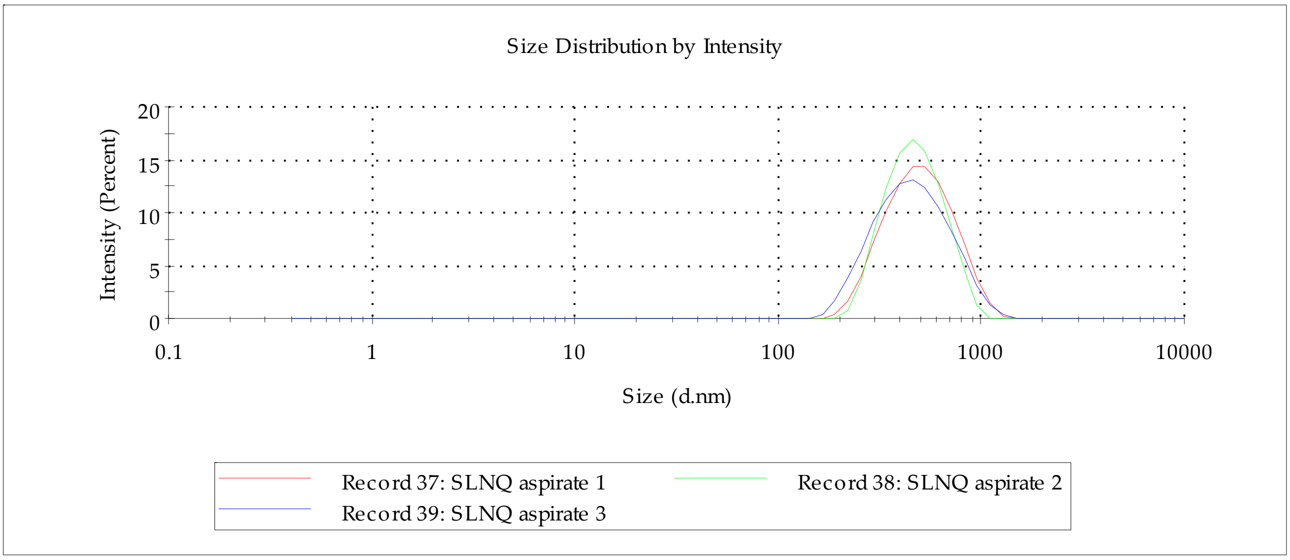

Figure S5: Size distribution for recovered from filter Quercetin-loaded Solid Lipid Nanoparticles

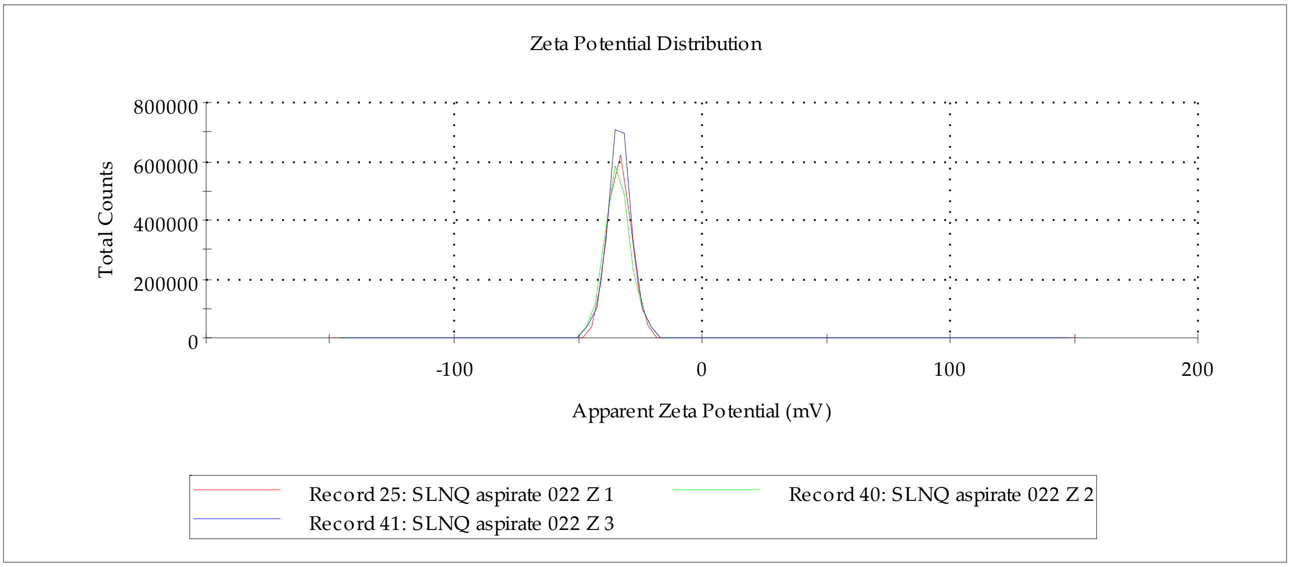

Figure S6: Zeta potential distributions for recovered from filter Quercetin-Loaded Solid Lipid Nanoparticles

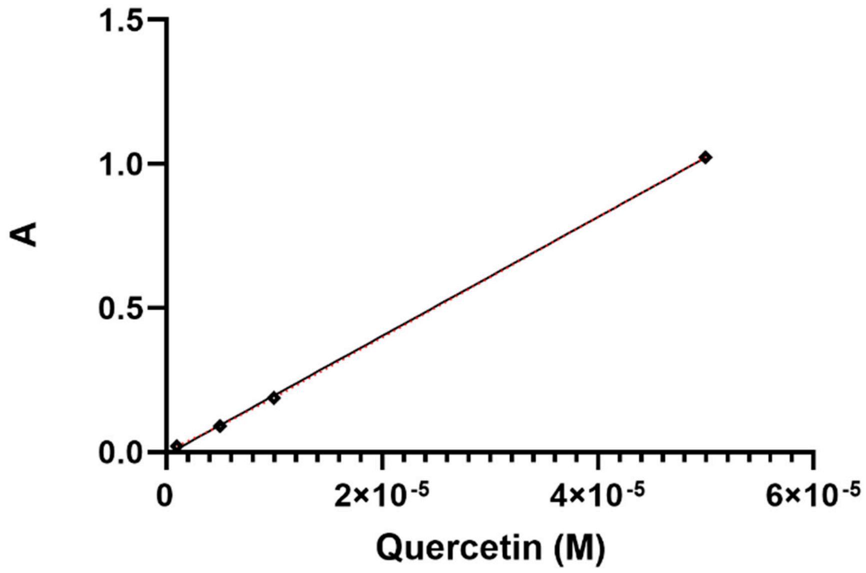

Figure S7: Calibraton curve for Quercetin in EtOH. R^2=0.9997, eq. Y = 20630X - 0.009693.
